# Supplementary material for: Are Routine Chest X-rays Necessary following Thoracic Surgery? A Systematic Literature Review and Meta-Analysis
Source: Cancers (Basel). 2022 Sep 7;14(18):4361. doi: 10.3390/cancers14184361 (PMC9496662; doi:10.3390/cancers14184361)
Supplement: Supplementary file 1 [file cancers-14-04361-s001.zip › LCO_Search Strategie Full.pdf]

Appendix 1. Search strategies

MEDLINE (via PubMed)

#1 "Thoracic Surgical Procedures"[MeSH Terms:noexp] OR "Thymectomy"[MeSH Terms] OR "Thoracotomy"[MeSH Terms] OR "Thoracostomy"[MeSH Terms] OR "Thoracoscopy"[MeSH Terms] OR "Thoracoplasty"[MeSH Terms] OR "Sternotomy"[MeSH Terms] OR "Pulmonary Surgical Procedures"[MeSH Terms] OR "Mediastinoscopy"[MeSH Terms]

#2 "thymectom*"[Title/Abstract] OR "thoracotom*"[Title/Abstract] OR "thoracostom*"[Title/Abstract] OR "pleuroscop*"[Title/Abstract] OR "thoracoscop*"[Title/Abstract] OR "thoracoplast*"[Title/Abstract] OR "sternotom*"[Title/Abstract] OR "mediastinoscop*"[Title/Abstract] OR "pulmonary surger*"[Title/Abstract] OR "lung surger*"[Title/Abstract] OR "thorax surger*"[Title/Abstract] OR "thoracic surger*"[Title/Abstract]

#3 #1 OR #2

#4 "radiography, thoracic"[MeSH Terms:noexp] OR "Mass Chest X-Ray"[MeSH Terms]

#5 "thorax x ray*"[Title/Abstract] OR "thoracic x ray*"[Title/Abstract] OR "chest xray*"[Title/Abstract] OR "chest x ray*"[Title/Abstract]

#6 #4 OR #5

#7 #3 AND #6

#8 ("adolescen*"[Title/Abstract] OR "child*"[Title/Abstract] OR "schoolchild*"[Title/Abstract] OR "infant*"[Title/Abstract] OR "girl*"[Title/Abstract] OR "boy"[Title/Abstract] OR "boys"[Title/Abstract] OR "teen"[Title/Abstract] OR "teens"[Title/Abstract] OR "teenager*"[Title/Abstract] OR "youth*"[Title/Abstract] OR "pediatr*"[Title/Abstract] OR "paediatr*"[Title/Abstract] OR "puber*"[Title/Abstract] OR "infant"[MeSH Terms] OR "child"[MeSH Terms] OR "adolescent"[MeSH Terms]) NOT ("adult*"[Title/Abstract] OR "man"[Title/Abstract] OR "men"[Title/Abstract] OR "woman"[Title/Abstract] OR "women"[Title/Abstract])

#9 #7 NOT #8

#10 "animals"[MeSH Terms] NOT "humans"[MeSH Terms]

#11 #9 NOT #10

= 4587

Cochrane Central Register of Controlled Trials (CENTRAL)

#1 MeSH descriptor: [Thoracic Surgical Procedures] this term only

#2 MeSH descriptor: [Thymectomy] explode all trees

#3 MeSH descriptor: [Thoracotomy] explode all trees

#4 MeSH descriptor: [Thoracostomy] explode all trees

#5 MeSH descriptor: [Thoracoscopy] explode all trees

#6 MeSH descriptor: [Thoracoplasty] explode all trees

#7 MeSH descriptor: [Sternotomy] explode all trees

#8 MeSH descriptor: [Pulmonary Surgical Procedures] explode all trees

#9 MeSH descriptor: [Mediastinoscopy] explode all trees

#10 {OR #1-#9}

#11 ((pulmonary OR lung OR thorax OR thoracic) NEAR/3 surger*):ti,ab,kw

#12 (thymectom* OR thoracotom* OR thoracostom* OR pleuroscop* OR thoracoscop* OR thoracoplast* OR sternotom* OR mediastinoscop*):ti,ab,kw

#13 #11 OR #12

#14 #10 OR #13

#15 MeSH descriptor: [Radiography, Thoracic] this term only

#16 MeSH descriptor: [Mass Chest X-Ray] explode all trees

#17 ((chest OR thora*) NEAR/3 (x-ray* OR xray* OR x ray*)):ti,ab,kw

#18 {OR #15-#17}

#19 #10 AND #18

= 32

Cochrane Databaqse of Systematic Reviews (DARE)

#1 MeSH descriptor: [Thoracic Surgical Procedures] this term only

#2 MeSH descriptor: [Thymectomy] explode all trees

#3 MeSH descriptor: [Thoracotomy] explode all trees

#4 MeSH descriptor: [Thoracostomy] explode all trees

#5 MeSH descriptor: [Thoracoscopy] explode all trees

#6 MeSH descriptor: [Thoracoplasty] explode all trees

#7 MeSH descriptor: [Sternotomy] explode all trees

#8 MeSH descriptor: [Pulmonary Surgical Procedures] explode all trees

#9 MeSH descriptor: [Mediastinoscopy] explode all trees

#10 {OR #1-#9}

#11 ((pulmonary OR lung OR thorax OR thoracic) NEAR/3 surger*):ti,ab,kw

#12 (thymectom* OR thoracotom* OR thoracostom* OR pleuroscop* OR thoracoscop* OR thoracoplast* OR sternotom* OR mediastinoscop*):ti,ab,kw

#13 #11 OR #12

#14 #10 OR #13

#15 MeSH descriptor: [Radiography, Thoracic] this term only

#16 MeSH descriptor: [Mass Chest X-Ray] explode all trees

#17 ((chest OR thora*) NEAR/3 (x-ray* OR xray* OR x ray*)):ti,ab,kw

#18 {OR #15-#17}

#19 #10 AND #18

= 0

Web of Science

#5 (#3) AND #4

#4 TS=((chest OR thora*) NEAR/3 (xray* OR x-ray*))

#3 (#1) OR #2

#2 (TS=((pulmonary OR lung OR thora*) NEAR/3 surger*)) AND TS=((thymectom* OR thoracotom* OR thoracostom* OR pleuroscop* OR thoracoscop* OR thoracoplast* OR sternotom* OR mediastinoscop*))

#1 TS=((pulmonary OR lung OR thora*) NEAR/3 surger*)

= 355

CINAHL

S12 S10 AND S11

S11 S1 OR S2

S10 S3 OR S4 OR S5 OR S6 OR S7 OR S8 OR S9

S9 TI ((pulmonary OR lung OR thora*) N3 surger*) OR AB ((pulmonary OR lung OR thora*) N3 surger*)

S8 TI (thymectom* OR thoracotom* OR thoracostom* OR pleuroscop* OR thoracoscop* OR thoracoplast* OR sternotom* OR mediastinoscop*) OR AB (thymectom* OR thoracotom* OR thoracostom* OR pleuroscop* OR thoracoscop* OR thoracoplast* OR sternotom* OR mediastinoscop*)

S7 (MH "Surgery, Lung")

S6 (MH "Thoracoscopy")

S5 (MH "Thoracostomy")

S4 (MH "Thoracotomy")

S3 (MH "Thoracic Surgery")

S2 TI ((thora* OR chest) N3 (x-ray* OR xray* OR "x ray*")) OR AB ((thora* OR chest) N3 (x-ray* OR xray* OR "x ray*"))

S1 (MH "Radiography, Thoracic")

= 741
