# Supplementary material for: Are Routine Chest X-rays Necessary following Thoracic Surgery? A Systematic Literature Review and Meta-Analysis
Source: Cancers (Basel). 2022 Sep 7;14(18):4361. doi: 10.3390/cancers14184361 (PMC9496662; doi:10.3390/cancers14184361)
Supplement: Supplementary file 1 [file cancers-14-04361-s001.zip › Registration Prospero.pdf]

To enable PROSPERO to focus on COVID-19 submissions, this registration record has undergone basic automated checks for eligibility and is published exactly as submitted. PROSPERO has never provided peer review, and usual checking by the PROSPERO team does not endorse content. Therefore, automatically published records should be treated as any other PROSPERO registration. Further detail is provided here.

Citation

Ioannis Karampinis, Christian Galata. Routine chest x-rays are not necessary following thoracic surgery. A systematic literature review and meta-analysis.. PROSPERO 2021 CRD42021287314 Available from: https://www.crd.york.ac.uk/prospero/display_record.php?ID=CRD42021287314

Review question

Are the routine x-rays in the perioperative window following thoracic surgery associated with a relevant change in the perioperative patient care?

Searches

MEDLINE

Cochrane Library

Web of Science Core Collection

CINAHL

PsycINFO via EBSCO

WHO ICTRPClinicalTrial.gov

Search strategy

https://www.crd.york.ac.uk/PROSPEROFILES/287314_STRATEGY_20211025.pdf

Types of study to be included

Any type of prospective and retrospective studies will be included. Case reports and reviews will not be considered for eligibility

Condition or domain being studied

Patients undergoing thoracic surgery

Participants/population

Inclusion criteria

1. Patients undergoing thoracic surgery and receiving an xray in the postoperative window

2. Studies must report outcome regarding associated changes in patient care

Exclusion criteria:

1. Studies on children

2. Studies in animals

3. Studies involving patients undergoing cardiac surgery

4. Studies on patients on the intensive care unit or patients under mechanical ventilation

5. Unpublished studies, conference abstracts

Intervention(s), exposure(s)
X-ray in the postoperative phase following thoracic surgery

Comparator(s)/control
No routine x-ray in the postoperative phase following thoracic surgery

Main outcome(s)
Main outcome will be the x-ray related change in patient care. This will be interpreted as any diagnostic or therapeutic measure resulting as an immediate consequence to the xray.

Additional outcome(s)
Radiation exposure

Related costs

Data extraction (selection and coding)
The results of the literature research will be delivered in endnote. Two independent reviewers will search the reference list (abstracts) and define the ones which suit the above mentioned eligibility criteria. These references will be assessed as full text along with their references, which will be hand-searched for further studies that might have skipped the literature research. Abstracts in languages other than english will be translated (if required). Data extraction will be performed from the eligible studies by the two reviewers independently.

Risk of bias (quality) assessment
Risk of bias assessment will be considered based on the studies that will be included. If the majority of the studies to be included are retrospective, no risk of bias assessment will be performed because retrospective studies are highly biased anyway. If several prospective studies are available for analysis the appropriate bias assessment tools will be used accordingly.

Strategy for data synthesis
The analysis will be performed using MedCalc.

First we will calculate the relative risks and will present them as Forest plot. Two models will be calculated, fixed and random. The heterogeneity of studies will be calculated using the I^2 index. Depending on the study heterogeneity and the results of the I^2 test, the suitable model will be chosen in order to take into consideration the study variability if necessary. An I^2 value of 0 - 25 % represents insignificant heterogeneity; > 25 % - 50 % low heterogeneity; > 50 % - 75 % moderate heterogeneity; and > 75 % high heterogeneity. Analyses with insignificant heterogeneity will be calculated using a fixed-effects model and with a low or moderate heterogeneity using a random-effects model. If analyses with high heterogeneity exist, a sensitivity analysis will be performed. The confidence intervals will be presented accordingly.

The publication bias will be assessed using the Begg's test. We will perform two different analyses, one only including the randomized controlled studies and one including all the available studies. We will also perform a subgroup analysis to include only the patients undergoing lung resections and the effect of the autologous blood pleurodesis in this specific group. We will calculate the pooled success rate in this subgroup. Using the results we will perform a sample size calculation for an upcoming randomized controlled trial

Analysis of subgroups or subsets
No subgroup analysis is planned

Contact details for further information
Ioannis Karampinis
jkarabinis@yahoo.com

Organisational affiliation of the review
University Medical Center Mainz

Review team members and their organisational affiliations

Assistant/Associate Professor Ioannis Karampinis. University Medical Center Mainz
Assistant/Associate Professor Christian Galata. University Medical Center Mainz

Type and method of review

Systematic review

Anticipated or actual start date

01 November 2021

Anticipated completion date

30 June 2022

Funding sources/sponsors

The study will be funded by institutional ressources

Conflicts of interest

Yes

Language

English

Country

Germany

Stage of review

Review Ongoing

Subject index terms status

Subject indexing assigned by CRD

Subject index terms

MeSH headings have not been applied to this record

Date of registration in PROSPERO

25 November 2021

Date of first submission

25 October 2021

Stage of review at time of this submission

**Stage**	**Started**	**Completed**
Preliminary searches	No	No
Piloting of the study selection process	Yes	Yes
Formal screening of search results against eligibility criteria	Yes	No
Data extraction	No	No
Risk of bias (quality) assessment	No	No
Data analysis	No	No

The record owner confirms that the information they have supplied for this submission is accurate and complete and they understand that deliberate provision of inaccurate information or omission of data may be

construed as scientific misconduct.

The record owner confirms that they will update the status of the review when it is completed and will add publication details in due course.

Versions

25 November 2021

25 November 2021
